# Two-photon AIE bio-probe with large Stokes shift for specific imaging of lipid droplets†
†Electronic supplementary information (ESI) available: Experimental section, NMR, mass and absorption, HOMO, LUMO, photophysical properties, cell viability, cell imaging, photostability, and two-photon excited fluorescence spectra of TPA-PI. See DOI: 10.1039/c7sc01400g


**DOI:** 10.1039/c7sc01400g

**Published:** 2017-05-18

**Authors:** Meijuan Jiang, Xinggui Gu, Jacky W. Y. Lam, Yilin Zhang, Ryan T. K. Kwok, Kam Sing Wong, Ben Zhong Tang

**Affiliations:** a Department of Chemistry , Hong Kong Branch of Chinese National Engineering Research Centre for Tissue Restoration and Reconstruction , HKUST Jockey Club Institute for Advanced Study , Institute of Molecular Functional Materials , Division of Biomedical Engineering , State Key Laboratory of Molecular Neuroscience , Division of Life Science , Hong Kong University of Science and Technology , Clear Water Bay , Kowloon , Hong Kong . Email: tangbenz@ust.hk; b Guangdong Provincial Key Laboratory of Brain Science , Disease and Drug Development , HKUST-Shenzhen Research Institute , No. 9 Yuexing 1st RD, South Area, Hi-tech Park, Nanshan , Shenzhen 518057 , China; c Department of Physics , Hong Kong University of Science and Technology , Clear Water Bay , Kowloon , Hong Kong; d Guangdong Innovative Research Team , SCUT-HKUST Joint Research Laboratory , State Key Laboratory of Luminescent Materials and Devices , South China University of Technology , Guangzhou 510640 , China

## Abstract

Lipid droplets are dynamic organelles involved in various physiological processes and their detection is thus of high importance to biomedical research. Recent reports show that AIE probes for lipid droplet imaging have the superior advantages of high brightness, large Stokes shift and excellent photostability compared to commercial dyes but suffer from the problem of having a short excitation wavelength. In this work, an AIE probe, namely TPA-BI, was rationally designed and easily prepared from triphenylamine and imidazolone building blocks for the two-photon imaging of lipid droplets. TPA-BI exhibited TICT+AIE features with a large Stokes shift of up to 202 nm and a large two-photon absorption cross-section of up to 213 GM. TPA-BI was more suitable for two-photon imaging of the lipid droplets with the merits of a higher 3D resolution, lesser photobleaching, a reduced autofluorescence and deeper penetration in tissue slices than a commercial probe based on BODIPY 493/503, providing a promising imaging tool for lipid droplet tracking and analysis in biomedical research and clinical diagnosis.

## Abstract 2

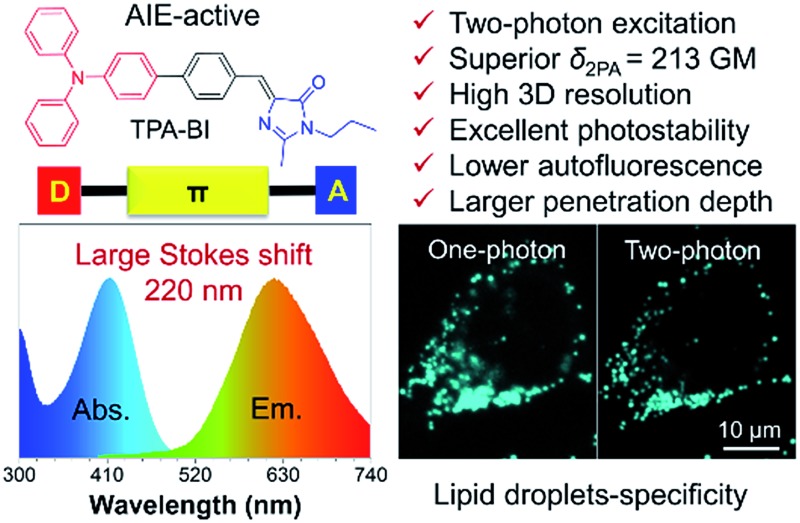
A novel AIEgen with prominent two-photon excitation was rationally developed for specific lipid-droplet imaging in cells and tissues.

## Introduction

Lipid droplets (LDs) that contain mainly diverse neutral lipids such as triacylglycerol and cholesteryl ester are widely found in adipocytes, hepatocytes and the adrenal cortex. For many years, LDs have been regarded as inert reservoirs of neutral lipids for energy storage. However, recent results show that LDs are considered to be dynamic organelles and associated with the storage and metabolism of lipids, signal transduction, apoptosis and so on.^1^ The abnormalities of LDs are generally related to some important diseases.^1^,^2^ For example, LDs are found to be critical for the proliferation of the hepatitis C virus,^3^ infection of which will lead to chronic hepatitis, liver cirrhosis and hepatocellular carcinoma. Thus, the localization and analysis of LDs are highly important for biomedical research and clinical diagnosis.

Techniques based on fluorescent materials are emerging as powerful and popular tools for biomedical studies both *in vitro* and *in vivo*.^4^ They exhibit excellent performances in applications such as localizing subcellular organelles, and monitoring the physiological changes of pH, temperature, viscosity, ions, proteins, and so on with the superior advantages of high resolution and sensitivity, easy operation and low cost. Recently, fluorescent probes for the localization of LDs have been developed. Two commercial dyes, namely Nile Red and BODIPY 493/503, are widely used but they show some fatal drawbacks, such as strong backgrounds and small Stokes shifts.^5^ Worse still, these conventional organic fluorophores unavoidably face a problem of aggregation-caused quenching (ACQ), where their fluorescence is quenched at high concentrations due to the formation of detrimental species such as excimers and exciplexes by strong π–π stacking.^6^ The ACQ effect has largely confined their working concentration to a very low nanomolar level, leading to quick photo-bleaching for bioimaging.

**Chart 1:**
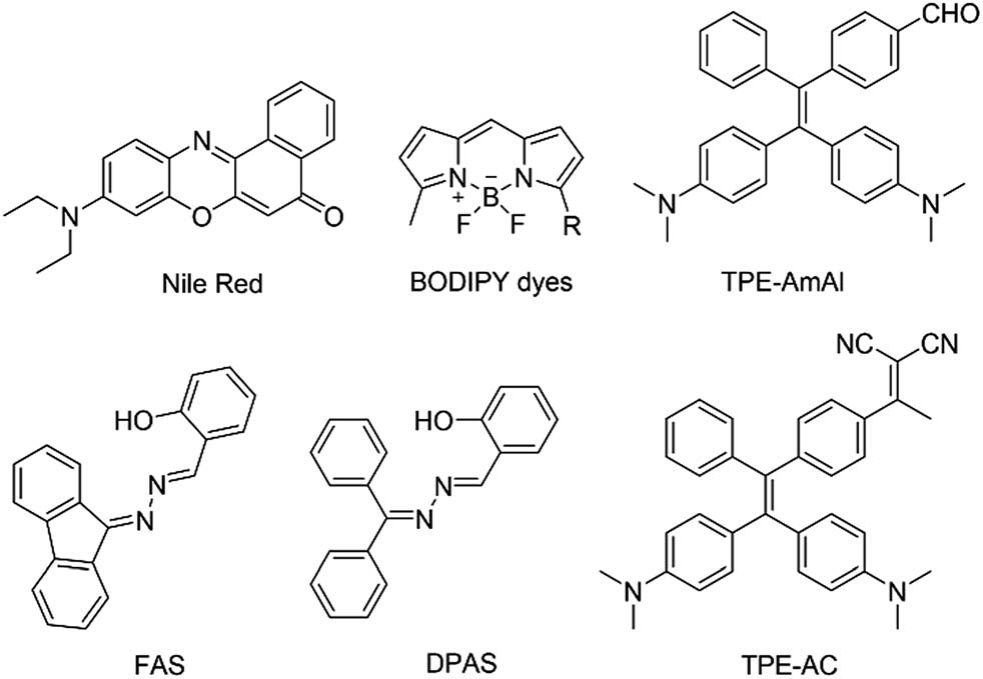
Chemical structures of lipid droplet imaging probes.

For many years, we and other groups have worked on the development of molecules with aggregation-induced emission (AIE) characteristics that are the exact opposite of the ACQ fluorophores. The restriction of intramolecular motion (RIM) has been proposed as the mechanism for the AIE effect.^7^ AIE luminogens (AIEgens) are weakly emissive in solutions due to the deactivation of the excited states by active intramolecular motions. However, such motions are suppressed in the aggregate state, thus enabling them to emit intensely upon excitation. AIEgens have found promising biomedical applications due to their superior merits of large Stokes shifts, high brightness, good biocompatibility, excellent photostability, *etc.*^8^ Therefore, the development of LD-specific AIE bioprobes could provide a promising approach to solving the problem observed in commercial dyes. Indeed, in our previous work, LD-specific AIE bioprobes, such as TPE-AmAl, FAS, DPAS and TPE-AC (Chart 1), show better performances in terms of brightness, specificity and photostability than their commercial counterparts in both fixed and living cell imaging. Meanwhile, these AIE-based bioprobes can be easily synthesized and have good cell permeability.^9^ However, most of the LD-specific AIE bioprobes developed so far bear either UV excitation or short-wavelength emission, which is harmful to living cells and suffers from limited penetration depth to tissue and serious auto-fluorescence from biosamples.^4^,^10^ Although TPE-AC exhibited a fascinating NIR emission (705 nm),^11^ the excitation wavelength was merely 450 nm, which was not long enough to reach the optical window for optimal tissue penetration (750–950 nm).10*b* Thus, LD-specific AIE bioprobes with excitation wavelengths in the red and near-infrared (NIR) regions will solve these problems and are in urgent demand. Many efforts have been devoted to designing new LD-specific AIE bioprobes with red and NIR excitations. Unfortunately, such a task is not easy in terms of tedious synthesis and low emission efficiency of the resulting molecules.

Recently, two-photon fluorescence microscopy (2PM) has become popular in biomedical diagnosis and therapy, due to its advantages of a longer-wavelength excitation, lower autofluorescence, higher 3D resolution and less photobleaching.^12^ Luminescent materials with two-photon excitation are crucially determined by a two-photon absorption (2PA) cross section (*δ*_2PA_). Materials bearing higher *δ*_2PA_ will show stronger two-photon excited fluorescence (TPEF) and a less deleterious thermal effect from the strong laser pulse.^13^ Therefore, the design of AIE bioprobes with two-photon excitations can provide an easier way to realize red and NIR excitations.

**Scheme 1:**
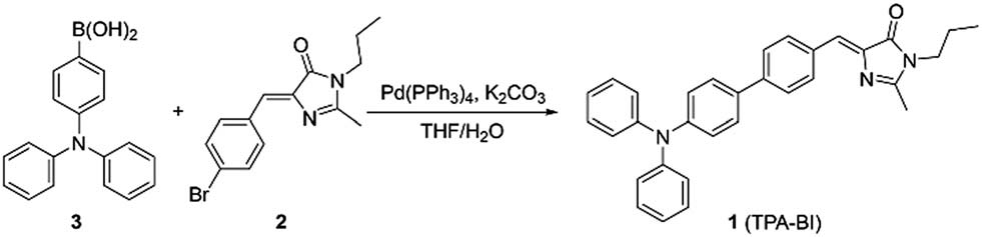
Synthetic route to TPA-BI.

Benzylidene imidazolone (BI), the analogue of the chromophore of green fluorescent protein, has been wildly-studied due to its facile synthesis and excellent biocompatibility.^14^ Recently, many of its derivatives have been designed and found to be AIE-active.^15^ Compared to TPE, BI possesses a more rigid structure with a less twisted conformation and would be an ideal building block for 2PA materials.^16^ However, to the best of our knowledge, BI-based 2PA materials have been rarely reported. Herein, we attempt to integrate the merits of AIE and 2PA into BI. On the other hand, triphenylamine (TPA) is a popular design unit for 2PA10b,^17^ and a well-known strong electron donor. The decoration of BI with TPA is thus expected to give a luminogen with a high *δ*_2PA_ and a longer-wavelength emission. The structural design of the molecule, abbreviated to TPA-BI, is shown in Scheme 1. Indeed, TPA-BI possessed a large *δ*_2PA_ and exhibited strong TPEF. TPA-BI can specifically stain lipid droplets in both fixed and live cells with a large Stokes shift and a superior two-photon imaging performance.

## Results and discussion

### Synthesis

TPA-BI was readily synthesized in a good yield by the Suzuki coupling of (*Z*)-5-(4-bromobenzylidene)-2-methyl-3-propyl-3,5-dihydro-4*H*-imidazol-4-one (**2**) and (4-(diphenylamino)phenyl)boronic acid (**3**) (Scheme 1). Detailed experimental procedures are provided in the Electronic Supplementary Information (ESI†). The structure of TPA-BI was fully characterized and confirmed by NMR and high-resolution mass spectroscopies (ESI, Fig. S1–S3†).

### Solvatochromism and twisted intramolecular charge-transfer

**Fig. 1:**
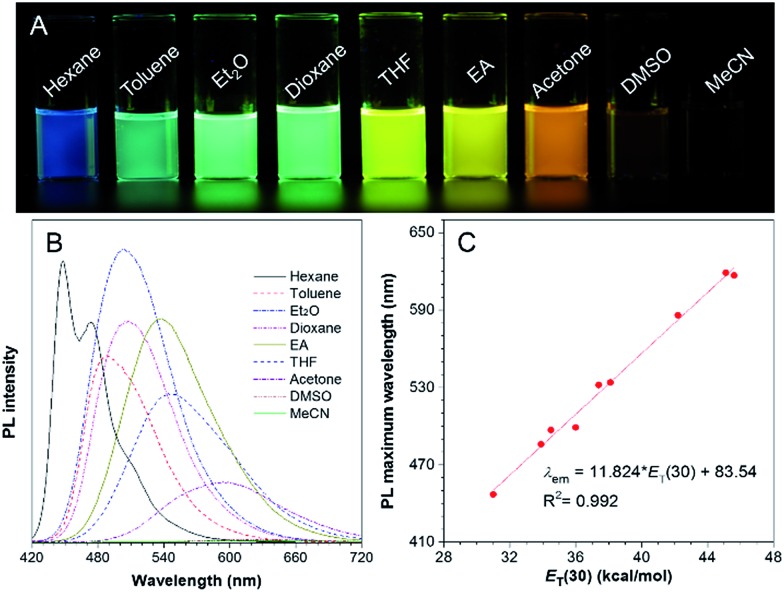
(A) Photographs of TPA-BI in different solvents taken under 365 nm UV irradiation from a hand-held UV lamp. (B) PL spectra of TPA-BI in different solvents. (C) Plot of the emission maximum of TPA-BI in different solvents *versus E*_T_(30), where *E*_T_(30) was the empirical parameter for solvent polarity. Concentration: 10 μM; *λ*_ex_ = 380 nm.

Molecules with donor (D)–π–acceptor (A) structures are characterized by a prominent solvatochromic effect, where their photophysical properties change by varying the solvent polarity. Hence, the absorption and photoluminescence (PL) spectra of TPA-BI in solvents with different polarities were investigated and the results are shown in Fig. 1 and S4.† In Fig. 1A, under UV light irradiation, the emission colour of the TPA-BI solution could be finely tuned from blue to red when the solvent changed from *n*-hexane to acetonitrile, nearly covering the full visible spectrum. The emission maximum varied gradually from 447 nm to 619 nm (Fig. 1B). Simultaneously, a pronounced decrease in the emission intensity was observed. On the contrary, the absorption of TPA-BI exhibited little change on changing the solvent polarity (Fig. S4†). The absorption maximum of TPA-BI only changed from 400 nm to 414 nm by increasing the solvent polarity with an extinction coefficient of ∼34 000 M^–1^ cm^–1^. All of these results indicate that the photophysical properties of TPA-BI are strongly dependent on the solvent polarity, which is ascribed to the twisted intramolecular charge transfer (TICT) effect from the electron-donating TPA unit to the electron-accepting imidazolone functionality. A large Stokes shift of up to 212 nm was realized, largely avoiding the overlap of the absorption spectrum and emission spectrum. This property is highly demanded for fluorescence probes as it prevents the self-absorption or “inner-filter” effect to increase the signal to noise ratio for fluorescence imaging.

To evaluate the effect of the solvents on the PL of TPA-BI, the change in the PL maximum with the solvent polarity parameter (*E*_T_(30))^18^ is plotted in Fig. 1C and summarized in Tables S1 and S2.† A linear line with a correlation coefficient of *R*^2^ = 0.992 and a large slope of 11.8 was obtained, indicating the remarkable solvatochromism of TPA-BI. The solvatochromic properties of TPA-BI were also confirmed by the dependence of the fluorescence transition energy on the solvent orientation polarizability (Δ*f*′) according to the revised Lippert–Mataga equation for TICT molecules (Table S1 and Fig. S5†). Both results indicate that TPA-BI shows strong solvatochromism resulting from the TICT effect. The TICT effect of TPA-BI can be interpreted by density functional theory (DFT) calculations (Fig. S6†). The photoexcitation from the S_0_ to S_1_ state of TPA-BI involves a substantial intramolecular charge transfer (ICT) from TPA to the imidazolone unit. Since the donor and acceptor are linked *via* a freely rotatable single bond, the activation of the ICT process is likely accompanied by a significant molecular geometry change and the formation of a TICT state. The TICT state will be largely stabilized and populated in solvents with higher polarity, resulting in a red-shift in the emission band. The TICT effect is responsible for the solvatochromism of TPA-BI and the increase in the Stokes shift from non-polar to polar solvents. The decrease in the PL intensity in a polar solvent should be attributed to the rapid consumption of the energy of the TICT state through non-radiative relaxation pathways.^19^

### Aggregation-induced emission

**Fig. 2:**
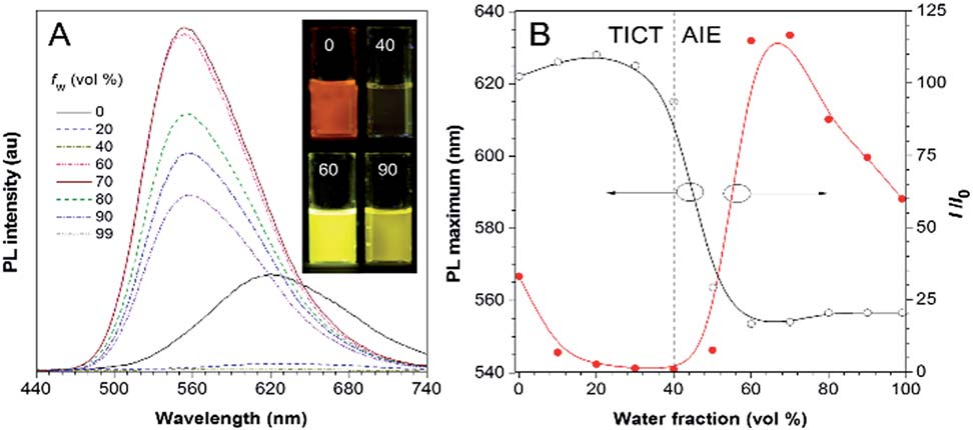
(A) PL spectra of TPA-BI in DMSO/water mixtures with different water fractions (*f*_w_). Inset: photographs of TPA-BI in DMSO/water mixtures with 0, 40, 60 and 90% water content taken in the presence of 365 nm UV irradiation from a hand-held UV lamp. (B) Plots of PL maximum and relative PL intensity (*I*/*I*_0_) *versus* the composition of the DMSO/water mixture of TPA-BI, where *I*_0_ was the PL intensity at 40% *f*_w_. Concentration = 10 μM; *λ*_ex_ = 380 nm.

Besides solvatochromism, TPA-BI also shows an aggregation-induced emission (AIE) phenomenon. As shown in Fig. 2, with an increase in the water fraction from 0 to 40% in the dimethylsulfoxide (DMSO)/water mixture, the emission of TPA-BI decreased, accompanied with a slight red-shift in the PL spectrum. This is due to the enhancement of the TICT effect in the presence of the more polar solvent of water in the surrounding environment. Upon further increasing the water fraction from 40% to 70%, an abrupt increase in the emission intensity (∼100-fold) was observed along with a blue shift in the PL maximum from 615 nm to 555 nm. To have a more accurate evaluation of the AIE characteristics, we have measured and plotted the quantum yields of TPA-BI in mixtures with different water fractions by an integrating sphere.^20^ The plot shows a similar trend with *I*/*I*_0_ (Fig. S7A†). The fluorescence quantum efficiency of TPA-BI in a 70% aqueous mixture was 22%, which was appreciably high for an orange emitter. Due to its poor solubility in water, in solution with high water fractions, aggregates of TPA-BI would be formed. This greatly restricts the intramolecular motion and activates the AIE process. The domination of the AIE effect over the TICT effect results in an increase in the PL intensity.9a,^11^ Surprisingly, the emission became weaker and was slightly red-shifted again when the water fraction increased from 70% to 90%. This may be attributed to (1) the crystallization-induced emission feature of TPA-BI and (2) the effect of the aggregate size.^21^ TPA-BI may form crystalline aggregates at low water fractions. At water fractions above 70%, the fast aggregation of the TPA-BI molecules will form less emissive, redder amorphous species with smaller sizes, as confirmed by the DLS results (Fig. S7B†). The emission of small-sized aggregates may be more vulnerable to being affected by the surrounding solvent environment, leading to an emission drop and red-shift at high water fractions.

### Two-photon excited fluorescence

**Fig. 3:**
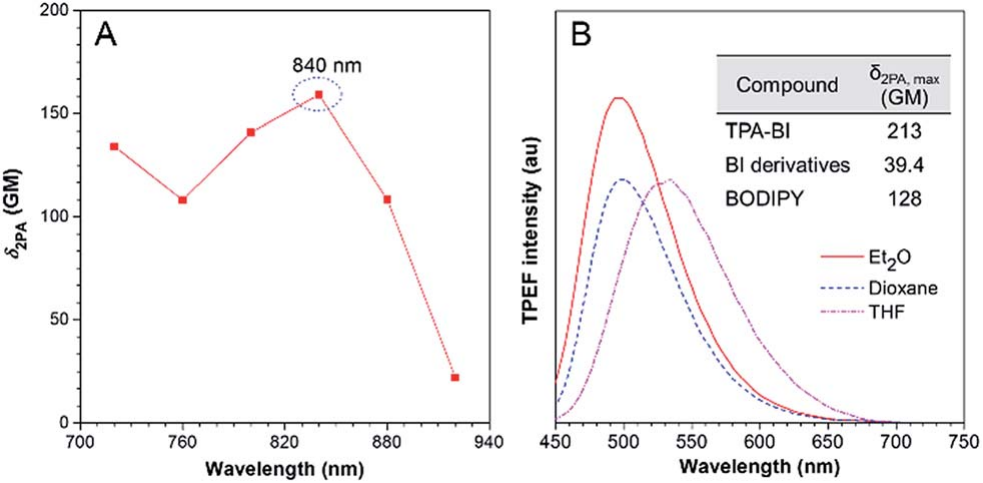
(A) Two-photon absorption (2PA) of TPA-BI in THF solution. (B) TPEF spectra of TPA-BI (40 μM) in different solvents excited by a Ti:Sapphire laser with 840 nm irradiation.

TPA-BI possesses a conjugated structure with strong electron donating and withdrawing groups and thus it is expected to exhibit strong 2PA. The 2PA of TPA-BI was studied using a TPEF technique with a femtosecond pulsed laser source, and the relative TPEF intensity in different solvents was measured using Rhodamine 6G and fluorescein as the standards.^22^ The measured wavelength was varied from 720 to 920 nm at an interval of 40 nm and the *δ*_2PA_ values were obtained. The results are summarized in Fig. 3 and Table S2.† In THF, the maximum *δ*_2PA_ value (159 GM) was obtained at 840 nm. In various solvents, the highest *δ*_2PA_ was obtained in diethyl ether and was equal to 213 GM, which was much higher than those of most fluorescent proteins (usually < 100 GM, only 39 GM for EGFP),^23^ synthetic BI derivatives (<40 GM),15*c* and BODIPY dyes (82–128 GM).^24^ Thus, TPA-BI may serve as a good two-photon imaging probe to living cells.

Apart from 2PA, the TPEF of TPA-BI under different laser powers was also studied. The plot of the fluorescence intensity against the excitation laser power gave a linear line with a slope of 1.911, confirming the occurrence of two photon absorption (Fig. S8†).19*c* When excited by laser light at 840 nm, TPA-BI emitted intense PL at 447–619 nm in solvents with different polarities, suggesting the TICT feature even under the condition of two-photon excitation (Table S2†). The spectral patterns resemble the one-photon ones, revealing the same excited state for the radiative decay processes (Fig. 3B). The TPEF cross sections (*δ*_2PEF_) are crucial parameters for biomedical imaging and are provided in Table S2.† The high *δ*_2PEF_ values in different solvents suggest that TPA-BI possesses a promising potential application in the biomedical field.

### One-photon LD imaging

To explore the application of TPA-BI in living cell imaging, its cytotoxicity was firstly evaluated using 3-(4,5-dimethyl-2-thiazolyl)-2,5-diphenyltetrazolium bromide (MTT) assay under different dye concentrations. As suggested in Fig. S9,† no significant variation in the cell viability was observed even when a high dye concentration of 20 μM was used. This indicates that TPA-BI shows almost no cytotoxicity to living cells and possesses a good cell biocompatibility.

**Fig. 4:**
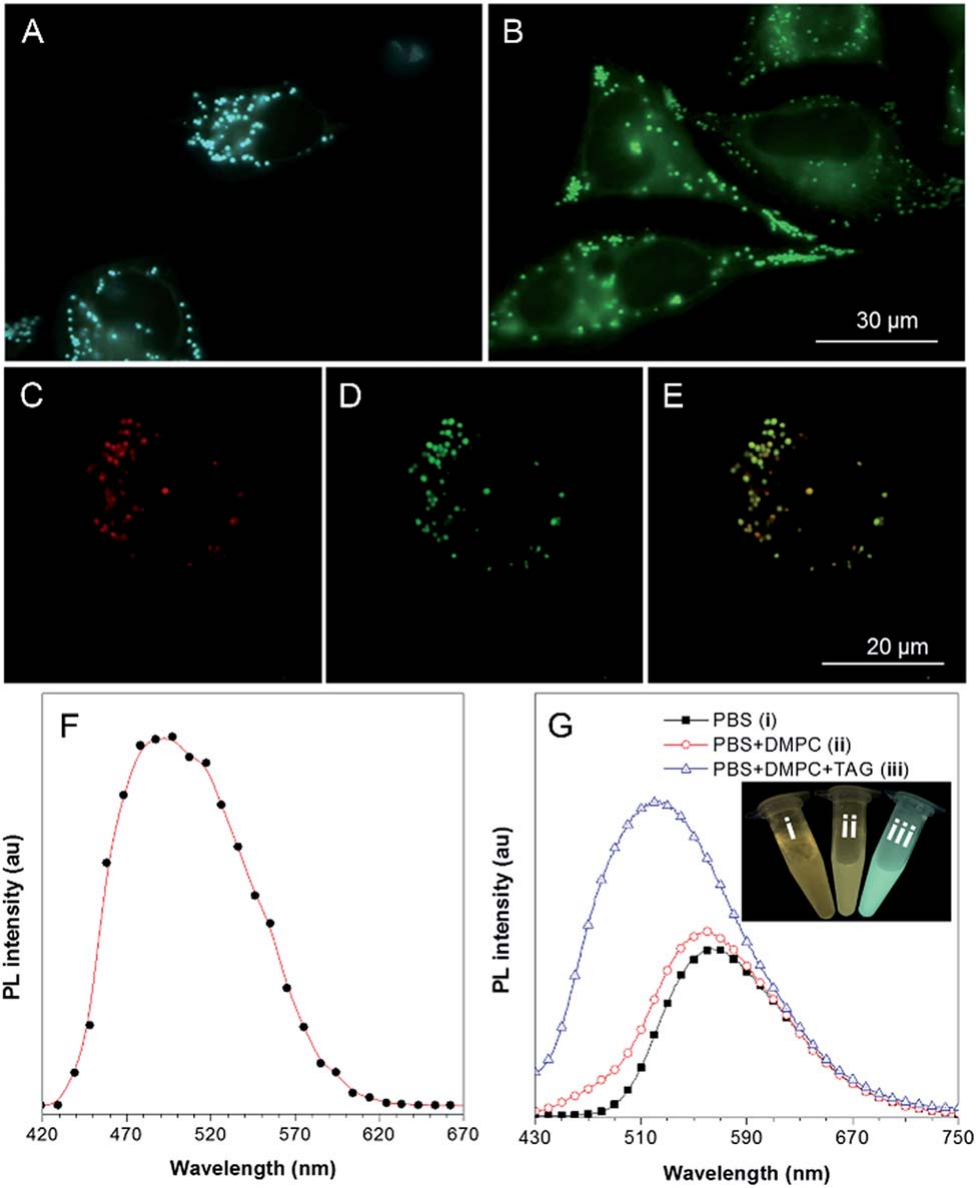
Fluorescence images of HeLa cells stained with (A) 1 μM TPA-MPI for 15 min and (B) 1 μg mL^–1^ (3.8 μM) BODIPY 493/503 for 15 min. *λ*_ex_ = 400–440 nm for TPA-BI and 460–490 nm for BODIPY 493/503. Scale bar: 30 μm. (C–E) Confocal images for the colocalization of TPA-BI with BODIPY 493/503: (C) image of TPA-BI with red pseudo color for clearance of overlapping (*λ*_ex_ = 442 nm, *λ*_em_ = 450–500 nm), (D) image of BODIPY 493/503 (*λ*_ex_ = 488 nm, *λ*_em_ = 500–550 nm) and (E) merged image of C and D. (F) Fluorescence spectrum of lipid droplets in HeLa cells stained with TPA-BI. *λ*_ex_ = 405 nm. (G) Fluorescence spectra of TPA-BI (20 μM) in solutions of PBS, PBS with DMPC, and PBS with DMPC and TAG. Inset: photos of the corresponding solutions taken under 365 nm UV irradiation.

Cell imaging experiments were then carried out by incubating HeLa cells with 1 μM of TPA-BI for 15 min followed by examination under a fluorescence microscope at an excitation wavelength of 400–440 nm. As shown in Fig. 4A, the lipophilic TPA-BI was prone to accumulating in the hydrophobic spherical LDs with bright greenish-blue emission due to the “like–like” interactions. Compared with BODIPY 493/503, a commercial probe for LD imaging, the images stained by TPA-BI showed a lower background signal, thanks to its AIE feature. Colocalization of TPA-BI and BODIPY 493/503 was performed and the same patterns were obtained solely by TPA-BI or BODIPY 493/503 with good overlap, demonstrating a good specificity of TPA-BI to LDs (Fig. 4C–E and S10†).

Besides a high LD specificity, TPA-BI also showed an excellent resistance to photo-bleaching. More than 80% of its fluorescence signal was retained even when it was continuously irradiated by laser light for 50 scans (Fig. S11†). Such a high photostability is comparable to that of BODIPY 493/503.^25^ TPA-BI can also be utilized in LD imaging in other cells lines, such as HepG-2 and A549, and in fixed cells (Fig. S12†). In addition, a negligible emission color change was observed with the increase of the dye concentration, oleic acid concentration or incubation time of oleic acid (Fig. S13 and S14F†). However, more and larger lipid droplets were observed after increasing the concentration or incubation time of oleic acid, and the fluorescence intensity of the whole cell was increased (Fig. S13 and S14A–E†). The statistical results were further confirmed by flow cytometry using BODIPY 493/503 and TPA-BI for staining (Fig. S15†), suggesting that TPA-BI can be practically applied in the quantitative analysis of LDs by flow cytometry. All of these results demonstrate that TPA-BI indeed acts as a superior probe for LD imaging and analysis bearing wide applications in biomedical research and clinical diagnosis.

Why does TPA-BI exhibit greenish-blue emission in LDs? To understand this, we measured its fluorescence using a confocal microscope in the mode of wavelength scanning. In Fig. 4F, the fluorescence spectrum exhibits a peak at 495 nm. The peak value reflects the value of *E*_T_(30) of the environment and suggests a low polarity inside the LDs. This is understandable as the LDs are surrounded by a phospholipid monolayer and consist of various neutral lipids such as triacylglycerol and cholesteryl ester.1*a* To further verify our claim, we carried out the analogue experiment outside cells using the major components in LDs such as 1,2-dimyristoyl-*sn*-glycero-3-phosphocholine (DMPC) and trioleate glycerol (TAG). Without DMPC and TAG, the aggregates of TPA-BI in phosphate buffered saline (PBS) solution emitted orange coloured light at 570 nm, while the emission colour and intensity blue-shifted and increased slightly upon the addition of DMPC only. Further addition of TAG resulted in an abrupt increase in the emission intensity and a peak maximum (Fig. 4G). This should be ascribed to the TICT effect of TPA-BI since TAG is more hydrophobic and less polar than DMPC. Under two-photon excitation, the fluorescence intensity of TPA-BI increased more than 10-fold upon the addition of TAG, and the extent of this was higher than that achieved by one-photon excitation (Fig. S16†). This suggests a larger signal to noise ratio for LD imaging by two-photon excitation to allow better contrast. Thus, two-photon excitation clearly outperforms in LD imaging.

### Two-photon LD imaging

**Fig. 5:**
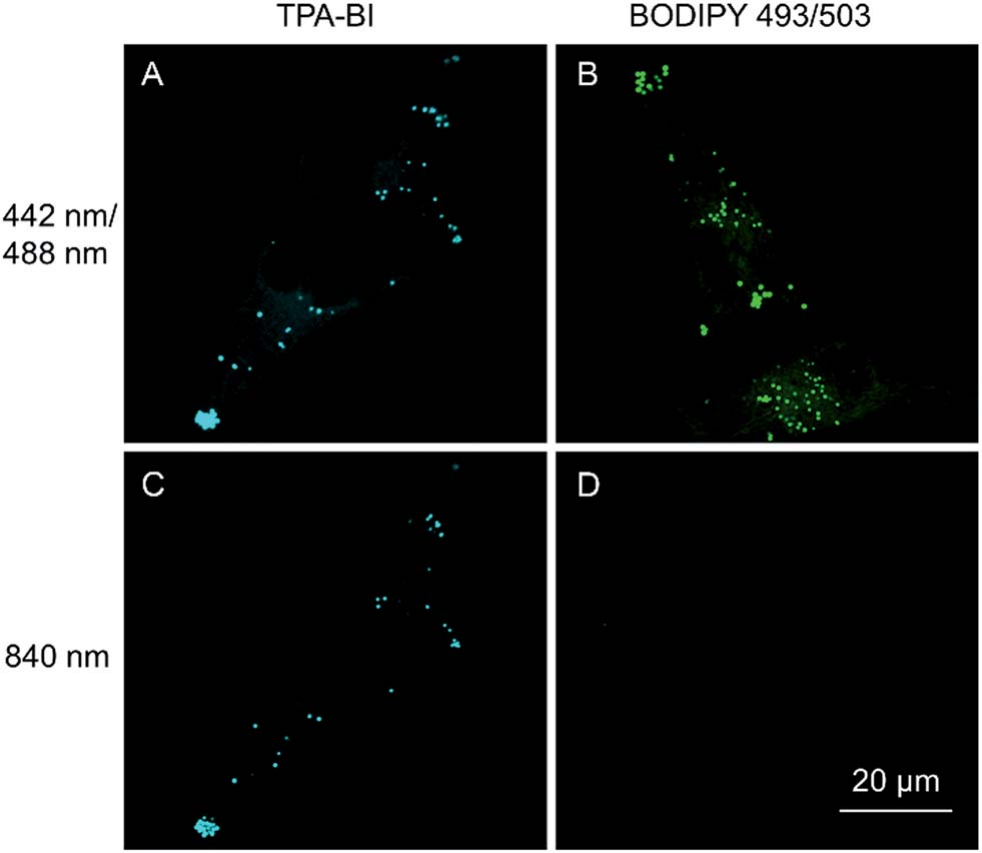
Confocal fluorescence images of HeLa cells stained with (A and C) 5 μM of TPA-BI and (B and D) 5 μM of BODIPY 493/503 for 20 min. Conditions: (A) *λ*_ex_ = 442 nm and *λ*_em_ = 450–550 nm, (B) *λ*_ex_ = 488 nm and *λ*_em_ = 500–600 nm, (C) *λ*_ex_ = 840 nm and *λ*_em_ = 450–550 nm, (D) *λ*_ex_ = 840 nm and *λ*_em_ = 500–600 nm.

As discussed above, TPA-BI shows a large *δ*_2PA_ of up to 213 GM and its response to the TAG/LDs is largely enhanced under two-photon excitation. To evaluate whether TPA-BI is suitable for two-photon imaging of the LDs, we compared its performance with commercial BODIPY 493/593. As seen in Fig. 5, sufficient signals were obtained for both TPA-BI and BODIPY 493/503 under one-photon excitation. While clear images of the LDs stained by TPA-BI were still observed at an excitation wavelength of 840 nm, almost no signal was obtained for the LDs stained by BODIPY 493/503 under the same conditions. Similar results were obtained even when the excitation wavelength was changed to 900 nm and 980 nm (Fig. S17†). Thus, TPA-BI is more suitable for two-photon imaging as it can be excited readily by laser light with a low power, thus avoiding photothermal damage to living cells caused by high laser power.

**Fig. 6:**
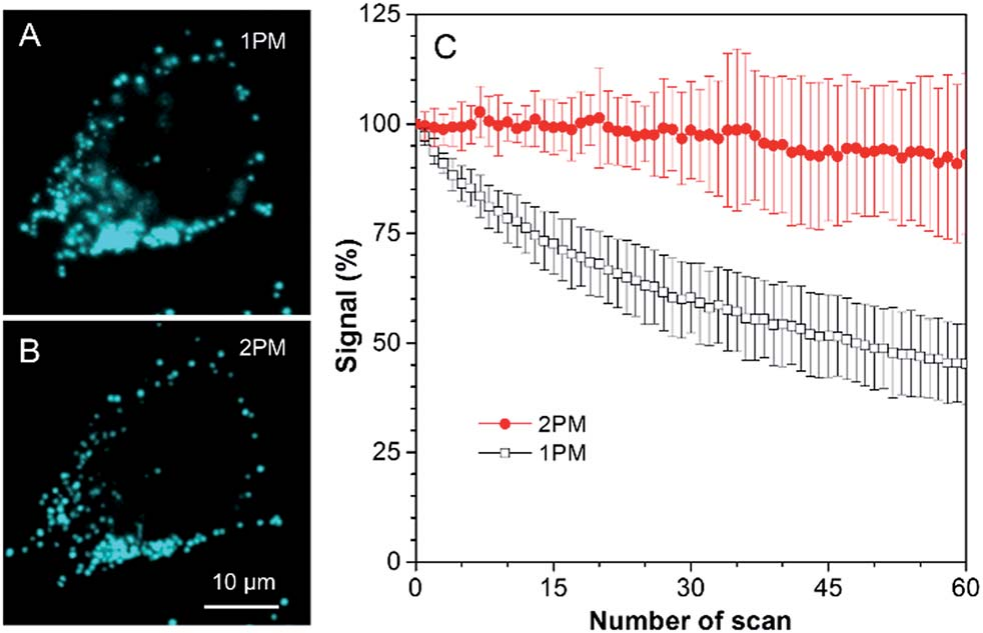
(A and B) Confocal images of HeLa cells stained with TPA-BI. HeLa cells were pre-treated with 50 μM oleic acid for 5.5 h. (C) Signal change in HeLa cells stained with TPA-BI upon continuous scanning by laser light. Concentration: 1 μM; *λ*_ex_ = 442 nm for (A) 1PM and 840 nm for (B) 2PM.

Several experiments were then conducted to demonstrate the superior advantages of two-photon microscopy (2PM) over one-photon microscopy (1PM) which are better 3D resolution, lesser photobleaching and autofluorescence and deeper penetration depth. As shown in Fig. 6A, clustered LDs in HeLa cells were observed with a blurred background by 1PM. The blurred background is believed to be caused by the fluorescence of the LDs below and above the focus plane. This problem was solved by 2PM due to the intrinsic sectioning property of 2PM. While a small layer of fluorophores was excited at the focus plane in 2PM, all of the fluorophores were excited in the light pathway in 1PM. Thus, fewer fluorophores were photobleached in 2PM during prolonged observation. To prove this, an experiment was carried at a low concentration of TPA-BI (1 μM) to enable the occurrence of photobleaching. As shown in Fig. 6B, while almost 100% of the signal intensity was retained in 2PM, only half was retained in 1PM.

**Fig. 7:**
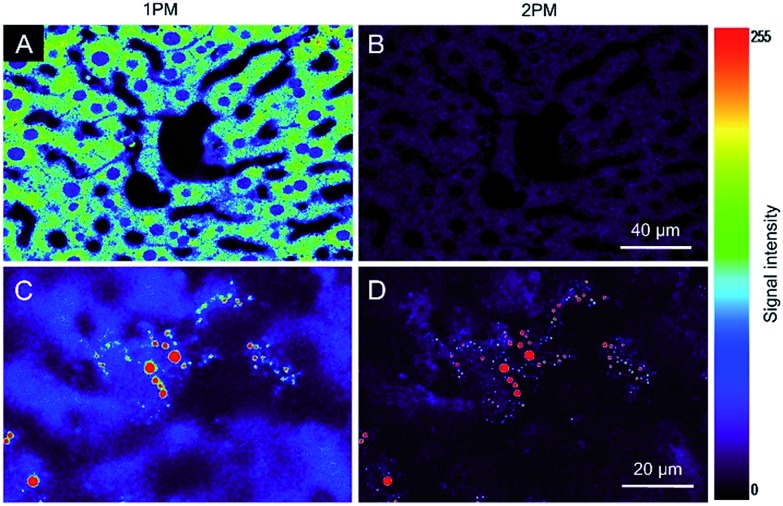
(A–D) Confocal images of fixed liver tissue slices stained (A and B) without and (C and D) with TPA-BI (10 μM) for 15 min at excitation wavelengths of (A and C) 442 nm and (B and D) 840 nm. The rainbow colour indicated the signal intensity. Scale bar: (A and B) 40 μm and (C and D) 20 μm.

**Fig. 8:**
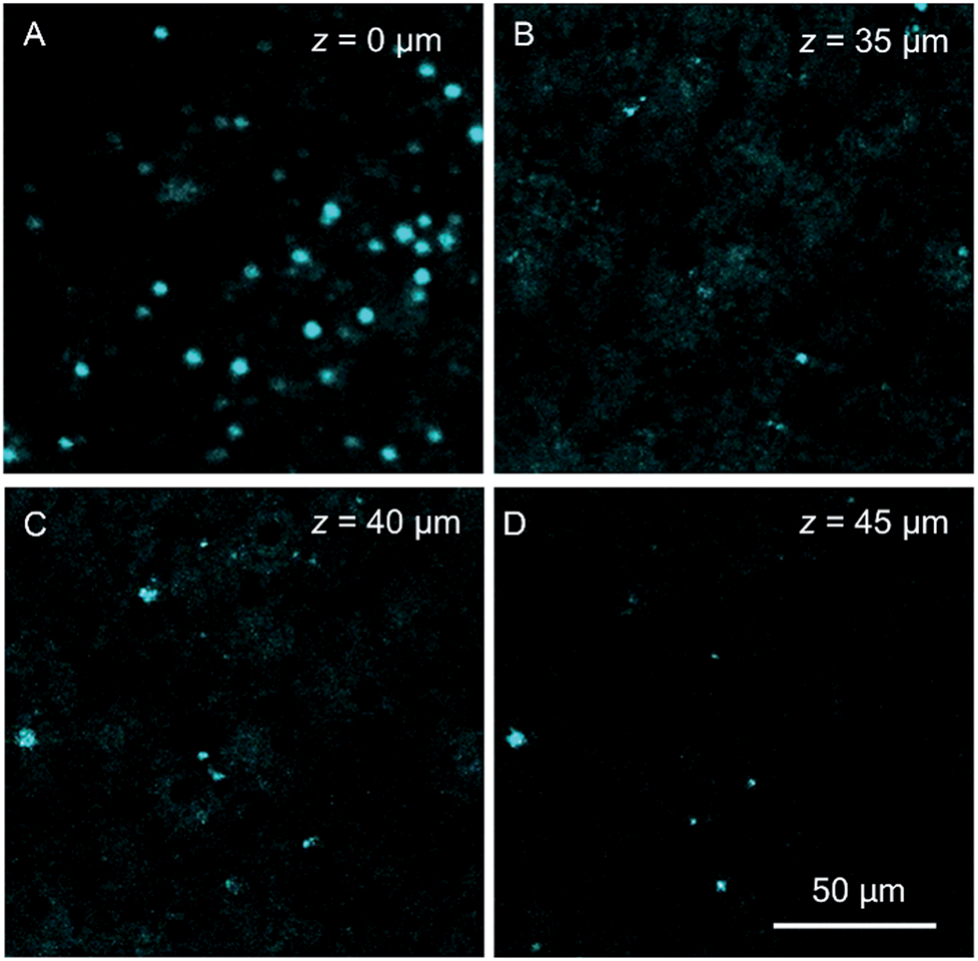
Confocal images of mice liver slices stained with TPA-BI (10 μM) at different penetration depths at an excitation wavelength of 840 nm.

Autofluorescence is a well-known difficult problem in tissue slices, which often leads to a low image contrast and is even detrimental to dyes with low emission intensity. Intense autofluorescence was observed in the fixed liver tissue slice by 1PM, which was largely reduced by 2PM (Fig. 7A and B). After staining with TPA-BI, clear spherical spots with intense fluorescence were observed with a much lower background than with 1PM (Fig. 7C and D). Due to the lesser absorption and scattering of the near-infrared light in the tissue,^26^ the longer excitation light (840 nm) in 2PM is believed to have a deeper penetration depth than that of one-photon excitation (442 nm). The fluorescent signal of the spherical spot could be detected at a *z* depth of 45 μm (Fig. 8). Compared to our previous LD-specific AIE bioprobes,^9^,^11^ TPA-BI not only exhibits the merits of AIE probes in 1PM but also performs well in 2PM with a large *δ*_2PA_ and NIR excitation, exhibiting higher 3D resolution, lower photobleaching rate, reduced auto-fluorescence and low damage to living cells. This makes TPA-BI suitable for LD imaging both in cells and tissue slices with two-photon excitation, providing another tool for tissue slice-based disease diagnosis of lipid droplets.

## Conclusion

In this work, an AIE probe (TPA-BI) for LD imaging was rationally designed and synthesized. Due to its D–π–A structure, TPA-BI exhibited solvatochromism with a high sensitivity to environmental polarity. TPA-BI exhibited both TICT and AIE features, showing a large Stokes shift of up to 202 nm and a large 2PA cross section of up to 213 GM. TPA-BI demonstrated good cell biocompatibility, high brightness, low background, high selectivity and excellent photostability. The lipid droplet imaging in TPA-BI was applicable for various live cell lines and fixed cells. It also allowed LD analysis by flow cytometry. Compared to commercial BODIPY dyes, TPA-BI was more suitable for two-photon imaging of LDs with the merits of higher 3D resolution, lesser photobleaching and autofluorescence and deeper penetration in tissue, providing a promising imaging tool for LD tracking and analysis in biomedical research and clinical diagnosis.

Due to its high sensitivity to polarity and good 2PA cross section, TPA-BI can be further utilized to detect the localized polarity of samples with two-photon excitation in a mixed bulk sample, such as for indicating the phase separation in polymer blends. Because of its synthetic accessibility, further modification of TPA-BI for imaging of other cell organelles or bio-sensing is under investigation in our laboratories.

## Supplementary Material

Supplementary information
