# Supplementary material for: Two-photon AIE bio-probe with large Stokes shift for specific imaging of lipid droplets
Source: Chem Sci. 2017 May 18;8(8):5440–6. doi: 10.1039/c7sc01400g (PMC5609514; doi:10.1039/c7sc01400g)
Supplement: Supplementary file 1 [file SC-008-C7SC01400G-s001.pdf]

Electronic Supplementary Material (ESI) for Chemical Science.
This journal is © The Royal Society of Chemistry 2017

Electronic Supplementary Information

Two-Photon AIE Bio-probe with Large Stokes Shift for Specific Imaging of Lipid Droplets

Meijuan Jiang,[ab#] Xinggui Gu,[ab#] Jacky W. Y. Lam,[ab] Yilin Zhang,[c] Ryan T. K. Kwok,[ab] Kam Sing Wong,[c] and Ben Zhong Tang[abd]

[a] *Department of Chemistry, Hong Kong Branch of Chinese National Engineering Research Centre for Tissue Restoration and Reconstruction, HKUST Jockey Club Institute for Advanced Study, Institute of Molecular Functional Materials, Division of Biomedical Engineering, State Key Laboratory of Molecular Neuroscience, Division of Life Science, Hong Kong University of Science and Technology, Clear Water Bay, Kowloon, Hong Kong. E-mail: tangbenz@ust.hk*

[b] *Guangdong Provincial Key Laboratory of Brain Science, Disease and Drug Development, HKUST-Shenzhen Research Institute, No. 9 Yuexing 1st RD, South Area, Hi-tech Park, Nanshan, Shenzhen 518057, China*

[c] *Department of physics, Hong Kong University of Science and Technology, Clear Water Bay, Kowloon, Hong Kong*

[d] *Guangdong Innovative Research Team, SCUT-HKUST Joint Research Laboratory, State Key Laboratory of Luminescent Materials and Devices, South China University of Technology, Guangzhou 510640, China*

[#] *Both authors contribute equally to the work.*

Experimental Section

Materials and Instrumentation: Chemicals for synthesis were purchased from J&K, Sigma-Aldrich and TCI, and used directly without further purification. Tetrahydrofuran (THF) was distilled from sodium/benzophenone under dry nitrogen immediately before use. Other solvents, such as dimethylsulfoxide (DMSO) were directly used without further purification. For cell culture, minimum essential medium (MEM), fetal bovine serum (FBS), penicillin-streptomycin solution and BODIPY 493/503 were purchased from Invitrogen. 3-(4,5-Dimethyl-2-thiazolyl)-2,5-diphenyltetrazolium bromide (MTT) were purchase from Aldrich. ^{1}H and ^{13}C-NMR spectra were recorded on a Bruker ARX 400 spectrometer using $CDCl_3$ as solvent and tetramethylsilane (TMS) as internal standard. High-resolution mass spectra (HRMS) were recorded on a GCT premier CAB048 mass spectrometer operated in a MALDI-TOF mode. UV-Vis absorption spectra were recorded on a Milton Roy Spectronic 3000 Array spectrameter. Photoluminescence spectra were recorded on a Perkin-Elmer LS 55 Spectrofluometer. Particle size analysis was determined at room temperature using a Zetaplus Potential Analyzer (Brookhaven Instruments Corporation, USA). All the solutions were measured in a 1 cm-thick quartz cell. Fluorescence quantum efficiency was measured by an integrating sphere. Two-photon excitation fluorescence cross section was

measured by two-photon excitation fluorescence method using rhodamine 6G and fluorescein as references.[1]

All the experiments, concerning the usage of fetal bovine serum and fixed mice liver slices, etc. were performed in compliance with the relevant laws and institutional guidelines. The institutional committee has approved the experiments.

Preparation of lipid and glyceryl trioleate solutions

Phosphate buffered solution (PBS)+1,2-dimyristoyl-sn-glycero-3-phosphocholine (DMPC, purchased from Avanti), PBS+DMPC+glyceryl trioleate (TAG, purchased from Sigma): 200 μL of 10mg/mL DMPC chloroform solution and 0 or 4 mg TAG, were added into an empty clean vial and the solvent was then removed under nitrogen. Afterwards, 5 mL of PBS was added and the solution was subjected to sonication for 10 min. 4 μL of 5 mM TPA-BI DMSO solution was added into 1 mL of the following solutions (PBS, PBS+ DMPC, PBS+DMPC+TAG). The resulting solutions were mixed by vortex. The final solution concentration was 20 μM for TPA-BI, 400 μg/mL for DMPC and 800 μg/mL for TAG.

Cell culture

HeLa cells were cultured in MEM containing 10% FBS and antibiotics (100 units per mL penicillin and 100 μg/mL streptomycin) in a 5% CO_2 humidity incubator at 37 °C.

Cell viability

Cells were seeded in 96-well plates at a density of 5000 cells per well. After culture for 24 h, the medium in each well was replaced by 100 μL of fresh medium containing different concentrations (0, 0.5, 1, 2.5, 5, 10 and 20 μM) of TPA-BI. The volume fraction of DMSO was below 0.2%. After 24 h, 10 μL of MTT solution (5 mg/mL in PBS) was added into each well. After 4 h incubation, 100 μL of SDS-HCl aqueous solution (10% SDS and 0.01 M HCl) was added to each well. After incubation for 6 h, the absorption of each well at 595 nm was recorded via a plate reader (Perkin-Elmer Victor3™). Each trial was performed with 6 wells parallel.

Cell treatment with oleic acid

HeLa cells were grown overnight on a 35-mm Petri dish with a cover slip. The cells were incubated with 50 μM of oleic acid for certain time (3−6 h) to induce lipid droplet formation.[2]

Cell imaging

HeLa cells were grown on a cover slip overnight in a 35-mm petri dish. The cells were stained with certain dye at certain concentration for certain time (by adding 2 μL of stock solution in DMSO to a 2 mL of culture medium with DMSO < 0.1 vol %). The cells were imaged under a fluorescent microscope (upright BX41 Microscope) using

---

[1] Y. Zhang, M. Jiang, G.-C. Han, K. Zhao, B. Z. Tang and K. S. Wong, *J. Phys. Chem. C*, 2015, **119**, 27630–27638.

[2] E. Wang, E. Zhao, Y. Hong, J. W. Y. Lam and B. Z. Tang, *J. Mater. Chem. B*, 2014, 2, 2013–2019.

proper excitation and emission filters for each dye: for TPA-BI, excitation filter = 400–440 nm, dichroic mirror = 455 nm, and emission filer = 465 nm long pass; for BODIPY, excitation filter = 460–490 nm, dichroic mirror = 505 nm and emission filter = 515 nm long pass.

Photostability

On a confocal microscope (Leica DMI 6000 Fully Motorized Inverted Microscope), the dyes were excited with 442 nm or 488 nm laser light for one-photon imaging and 840 nm for two-photon imaging. Imaging parameters were set for each dye individually to obtain optimal images. Repeated image scans were taken. On each series of scans, five/six regions of interest (ROIs) with several LDs were defined. The first scan of each ROI was set to 100%. Then the pixel intensity values for each ROI were averaged and plotted against the scan number. The resulting curve represented the bleaching rate that an experimentalist would encounter.

Flow cytometry

Six medium plates of HeLa cells were grown overnight. Then the cells were incubated with 50 μM of oleic acid for 0, 2, 3 and 4 h before collection for flow cytometry. The cells were stained with 1 μM of TPA-BI for 10 min and 1 μg/mL BODIPY for 15 min, respectively, and washed with PBS. The cells were analyzed by flow cytometry (Becton Dickinson FACS Aria IIIu). 10000 events were taken for each trial.

Synthesis of TPA-BI

TPA-BI (**1**) was synthesized following the synthetic route shown in Scheme 1. The key intermediates **2** and **3** were prepared according to Scheme S1 and the procedures described below.

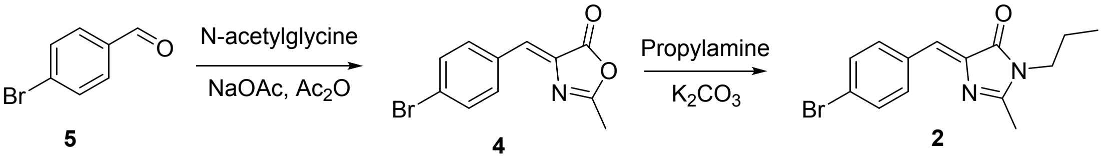

Scheme S1 Synthetic route to **1**.

Synthesis of **2**: 4-Bromo-benzylidene oxazolone (**4**) was synthesized by reaction of 4-bromobenzaldehyde (**5**) with *N*-acetylglycine using the procedure reported previously.[3] Briefly, a mixture of propylamine (320 mg, 5.4 mmol), 4-bromo-benzylidene oxazolone (1.34 g, 5 mmol) and potassium carbonate (60 mg) in 24 mL of THF/water mixture (1:1, v/v) was heated to reflux and stirred overnight. The solution was extracted with dichloromethane and the organic solvent was dried over $MgSO_4$. After removal of the solvent, the crude product was purified on a silica gel column with hexane/ethyl acetate mixture as eluent to afford the product as a white solid. Yield 54% (0.83g). ^1H

[3] G.-J. Huang and J.-S. Yang, *Chem. Asian J.*, 2010, **5**, 2075–2085.

NMR (400 MHz, $CDCl_3$) δ (ppm) 8.00 (d, 2H, J = 8.4 Hz), 7.54 (d, 2H, J = 8.4 Hz), 7.00 (s, 1H), 3.57 (t, 2H, J = 7.4 Hz), 2.39 (s, 3H), 1.69–1.62 (m, 2H), 0.96 (t, 2H, J = 7.4 Hz). ^{13}C NMR (100 MHz, $CDCl_3$) δ (ppm) 170.8, 163.3, 139.2, 133.6, 133.3, 132.1, 125.6, 124.7, 42.4, 22.8, 16.0, 11.4. MS (MALDI-TOF): calculated for $C_{14}H_{15}BrN_2O$: $[M]^+$ 306.0368, found 306.0367.

Synthesis of **3**: Compound **3** was synthesized according to the literature method. Briefly, to a solution of 4-bromotriphenylamine (3.2 g, 10 mmol) in freshly distilled THF was added 8 mL of BuLi (1.6 M in hexane) dropwise under nitrogen at -78 ^oC. After stirring for 2 h, 3.3 mL of trimethylborate was injected dropwise and the solution was warmed to room temperature and stirred for another 2 h. The reaction was quenched with dilute HCl solution. After solvent extraction and evaporation, the product was purified by silica gel column chromatography and obtained as white/grey solid. Yield 54% (1.57 g). ^1H NMR (400 MHz, $CDCl_3$) δ (ppm) 8.01 (d, 2H, J = 8.0 Hz), 7.31–7.28 (m, 4H), 7.17–7.05 (m, 8H).

Synthesis of **1**: Under nitrogen, **2** (307 mg, 1 mmol), **3** (289 mg, 1 mmol), $Pd(PPh_3)_4$ (40 mg), K_2CO_3 (210 mg) in 20 ml THF/water mixture (4:1, v/v) were heated to reflux overnight. After cooling to room temperature, the product was extracted with dichloromethane. After removal of the solvent, the crude product was purified on a silica gel column using hexane/ethyl acetate as eluent. Orange solid, yield 45% (210 mg). ^1H NMR (400 MHz, $CDCl_3$) δ (ppm) 8.18 (d, 2H, J = 8.0 Hz), 7.63 (d, 2H, J = 8.0 Hz), 7.52 (d, 2H, J = 8.4 Hz), 7.29 (d, 2H, J = 7.6 Hz), 7.15–7.13 (m, 7H), 7.05–7.03 (m, 2H), 3.59 (t, 2H, J = 7.4 Hz), 2.42 (s, 3H), 1.71–1.65 (m, 2H), 0.97 (t, 2H, J = 7.4 Hz). ^{13}C NMR (100 MHz, $CDCl_3$) δ (ppm) 170.9, 162.5, 148.0, 147.7, 142.2, 138.5, 134.0, 132.9, 132.8, 129.5, 127.9, 127.1, 126.9, 124.9, 123.7, 123.4, 42.4, 22.8, 16.0, 11.4. MS (MALDI-TOF): calculated for $C_{32}H_{29}N_3O$: $[M]^+$ 471.2311, found 471.2324.

NMR and MS spectra

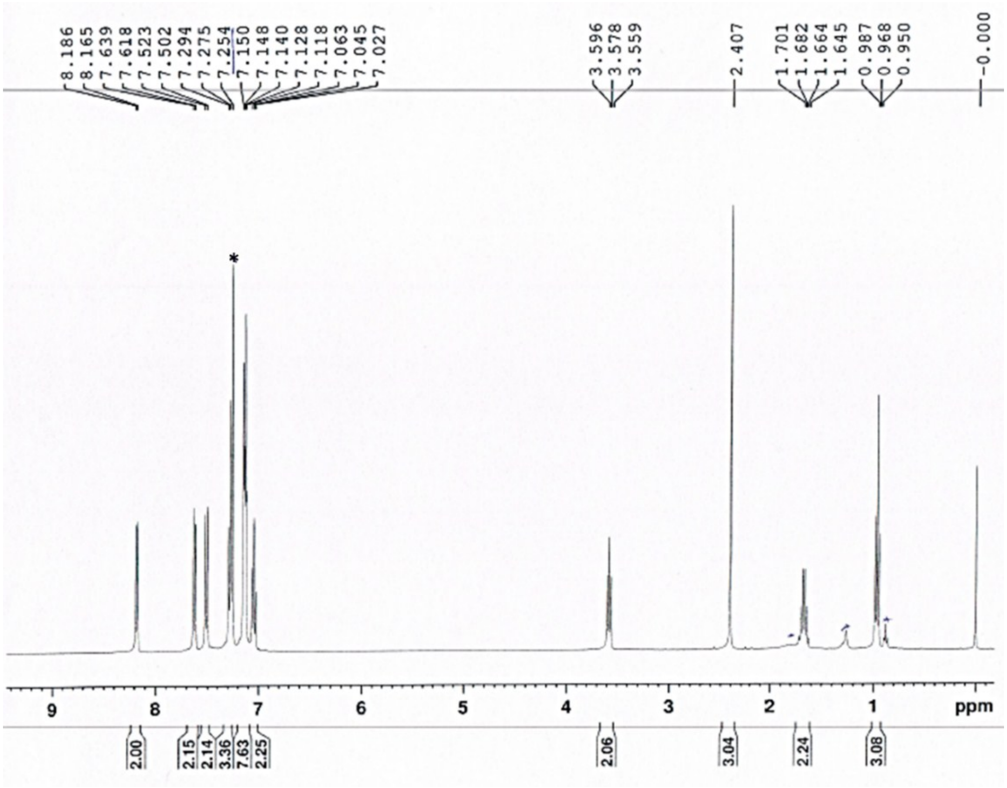

Fig. S1 ^{1}H NMR spectrum of TPA-BI in $CDCl_3$. The solvent peak was marked with asterisk.

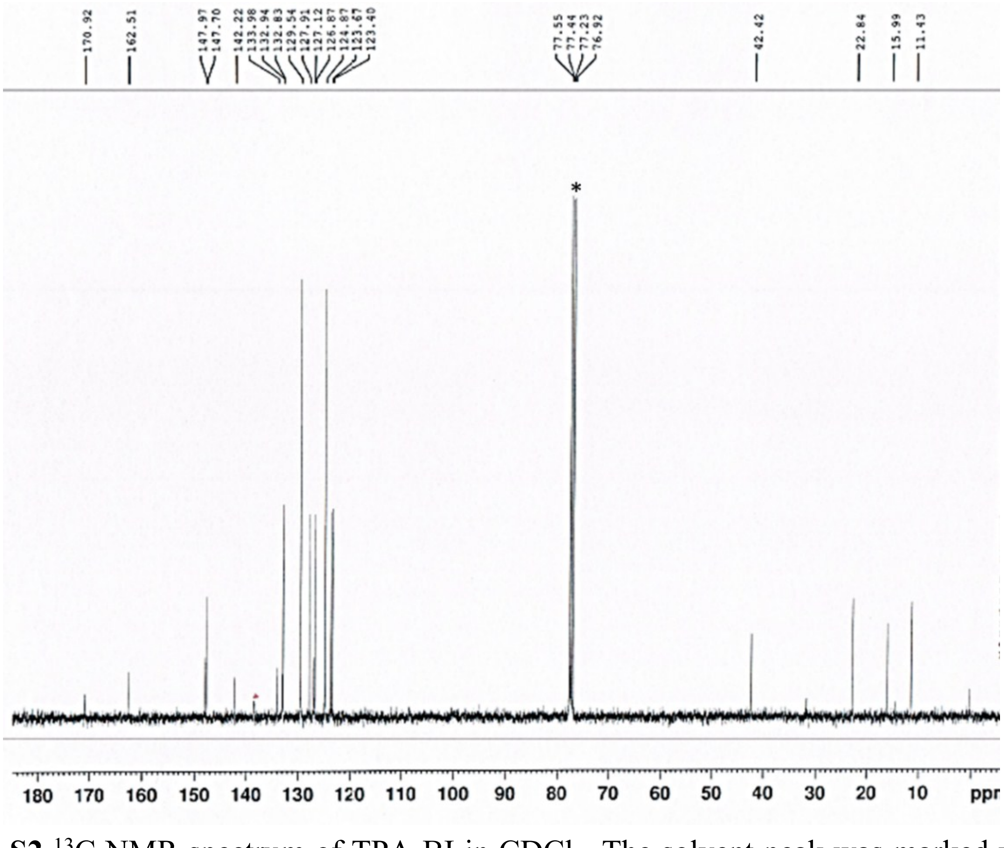

Fig. S2 ^{13}C NMR spectrum of TPA-BI in $CDCl_3$. The solvent peak was marked with

asterisk.

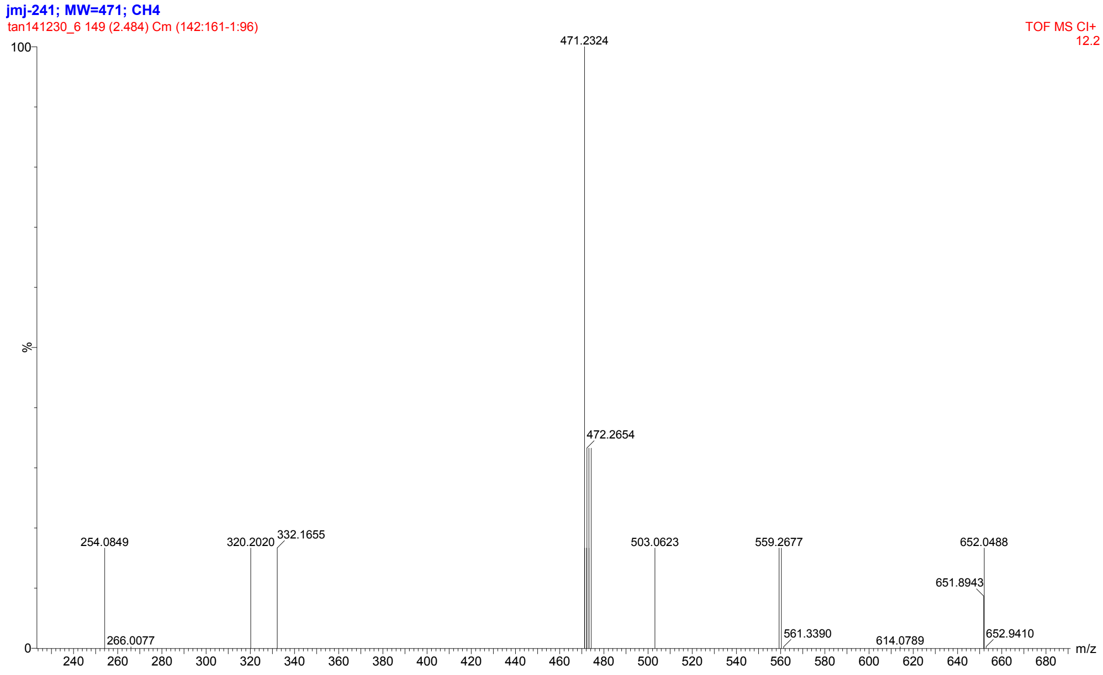

Fig. S3 High resolution mass spectrum of TPA-BI.

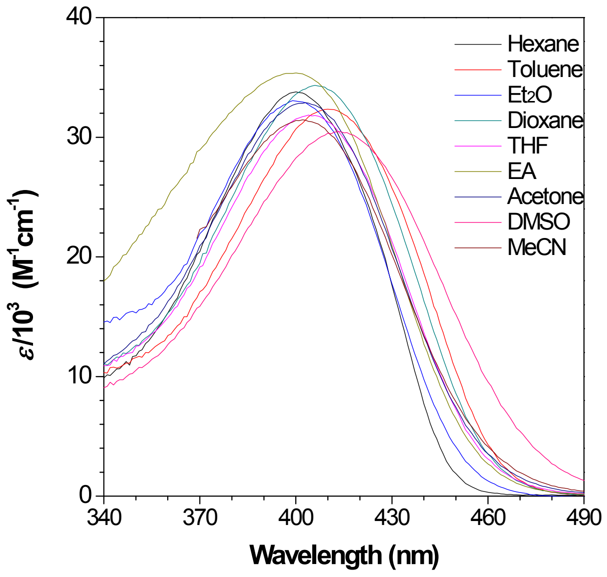

Fig. S4 Absorption spectra of TPA-BI in different solvents.

Table S1 Parameters of the solvents used [a]

solvent	ε	n	Δf	$\Delta f'$	$E_T(30)^e$ (kcal/mol)
Hexane	1.89	1.375	-0.001	0.113	31.0
Toluene	2.43	1.497	0.018	0.176	33.9
Et_2O	4.20	1.350	0.163	0.409	34.5
Dioxane	2.27	1.422	0.027	0.170	36.0
THF	7.47	1.402	0.210	0.561	37.4
EA	6.03	1.372	0.200	0.513	38.1
Acetone	21.36	1.359	0.285	0.761	42.2
DMSO	46.71	1.479	0.263	0.800	45.1
MeCN	35.94	1.344	0.305	0.815	45.6

[a] Abbreviation: ε = dielectric constant at 20 °C, n = refractive index at 20 °C, $\Delta f = \frac{\varepsilon - 1}{2\varepsilon + 1} - \frac{n^2 - 1}{2n^2 + 1}$ (orientation polarizability), $\Delta f' = \frac{\varepsilon - 1}{\varepsilon + 2} - \frac{n^2 - 1}{2n^2 + 4}$ (revised orientation polarizability for TICT effect) and $E_T(30)$ = empirical parameters for solvent polarity. The solutions were sorted in ascending order of $E_T(30)$.

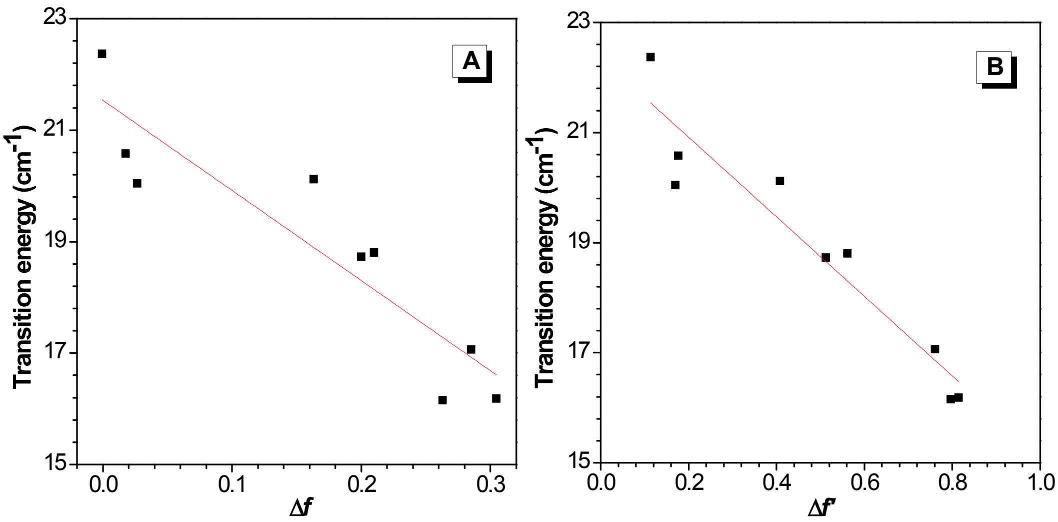

Fig. S5 Plots of fluorescence transition energy of TPA-BI versus (A) Δf (R^2 = 0.814) and (B) $\Delta f'$ (R^2 = 0.898).

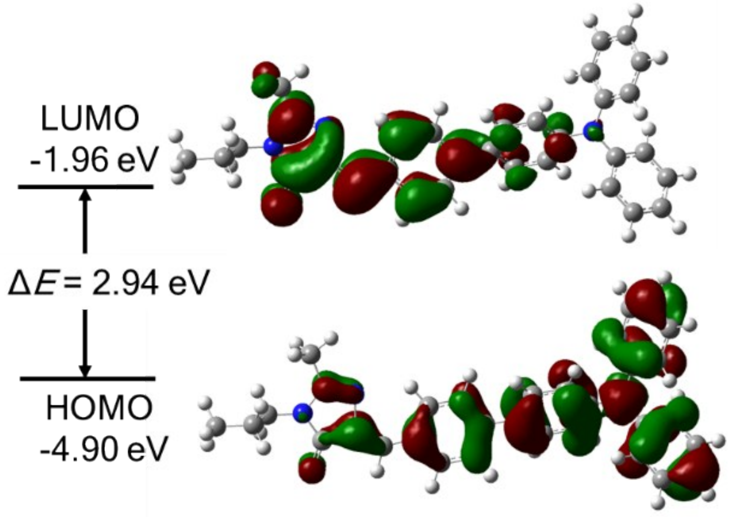

Fig. S6 HOMO and LUMO of TPA-BI by DFT calculations at the base level of B3LYP/6-31G via Gaussian 09 program.

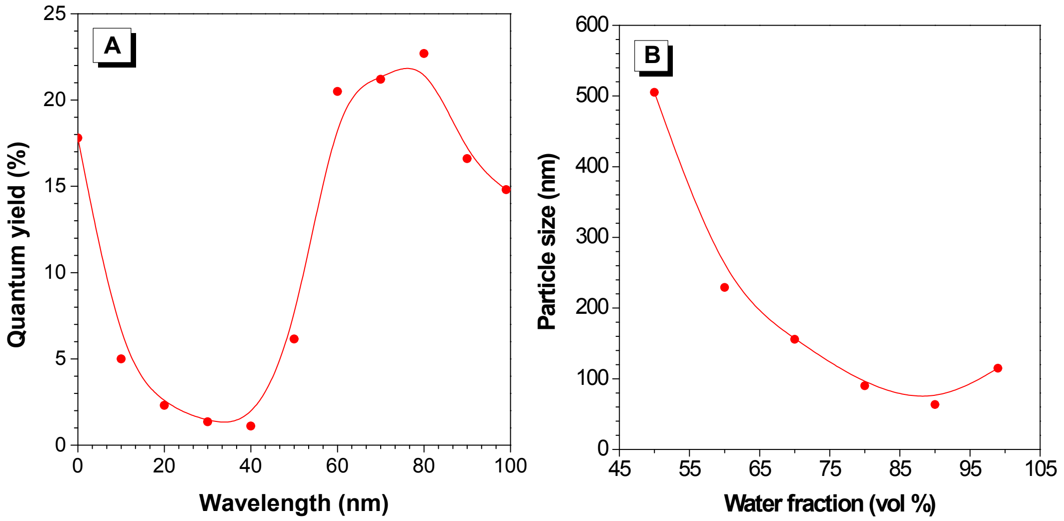

Fig. S7 (A) Quantum yield of TPA-BI and in DMSO/water mixtures with different water fractions measured by an integrating sphere. (B) Particle size of TPA-BI aggregates formed in DMSO/water mixtures with different water fractions. No obvious aggregates were detected in the mixtures with water fractions lower than 50 vol %. Concentration = 10 μM.

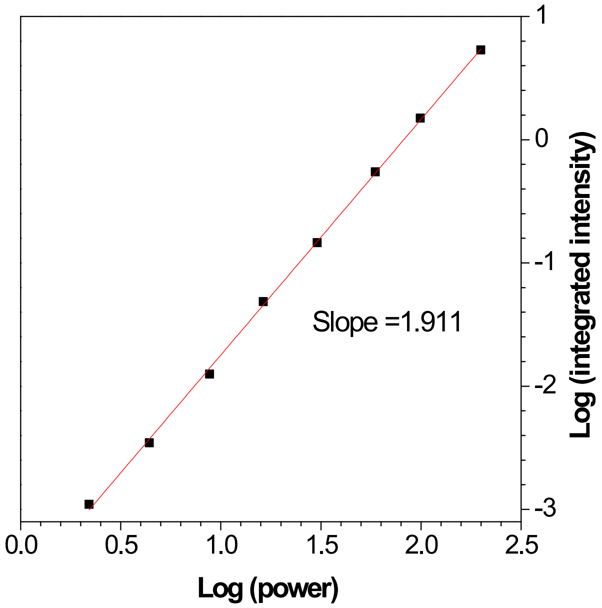

Fig. S8 Plot of logarithm integrated intensity of TPA-BI in THF solution versus logarithm laser power. Concentration = 40 μM; laser: Ti:Sapphire.

Table S2 Summarized photophysical data of TPA-BI. [a]

	λ_{abs} (nm)	λ_{em} (nm)	Stokes shift (nm)	Φ	δ_{2PEF} (GM)	δ_{2PA} (GM)
Hexane	400	447	47	0.17	28.7	164.3
Toluene	410	486	76	0.28	50.7	182.3
Et_2O	400	497	97	0.42	89.6	213.4
Dioxane	406	499	93	0.37	67.3	182.0
THF	404	532	128	0.61	97.1	157.9
EA	400	534	134	0.52	77.2	148.5
Acetone	403	586	183	0.32	38.5	121.0
DMSO	414	617	203	0.13	11.1	85.2
MeCN	402	619	217	0.04	3.5	82.0
Aggre.	416	570	154	0.22	16.9	76.8

[a] Abbreviation: λ_{abs} = absorption maximum, λ_{em} = emission maximum, Φ = fluorescence quantum yield, δ_{2PEF} = two-photon excitation fluorescence cross section, where 1 GM ≡ 10^{-50} cm^4 s/photon, δ_{2PA} = two-photon absorption cross section, aggre. = aggregates of TPA-BI in DMSO/water mixture with 60% water content. Concentration: 40 μM (solution) and 20 μM (aggregate suspension).

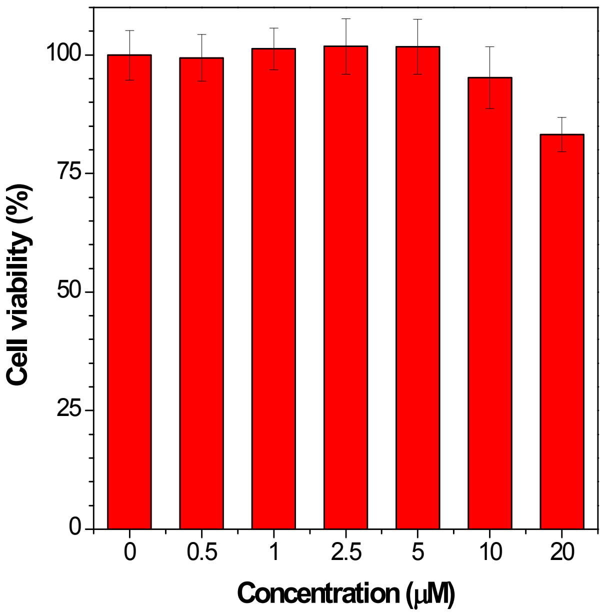

Fig. S9 Cell viability of HeLa cells in the presence of different concentrations of TPA-BI determined by MTT assay.

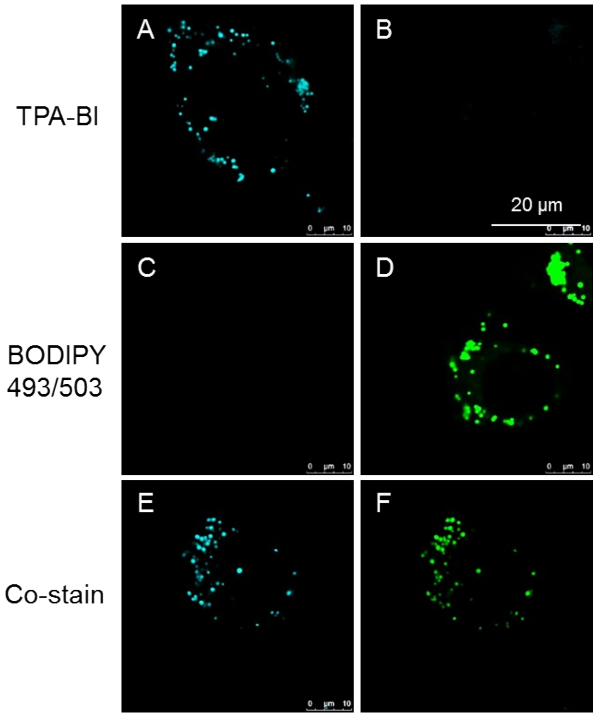

Fig. S10 Colocalization: confocal images of HeLa cells stained with (A and B) TPA-BI, (C and D) BODIPY and (E and F) both TPA-BI and BODIPY. Condition: (A, C and E) λ_{ex} = 442 nm, λ_{em} = 450-500 nm; (B, D and F) λ_{ex} = 488 nm, λ_{em} = 500-550 nm. Dye concentration: 5 μM for TPA-BI and 1μg/mL (3.8 μM) BODIPY 493/503. Incubation time: 15 min. The HeLa cells were pretreated with 50 μM of oleic acid for 5.5 h.

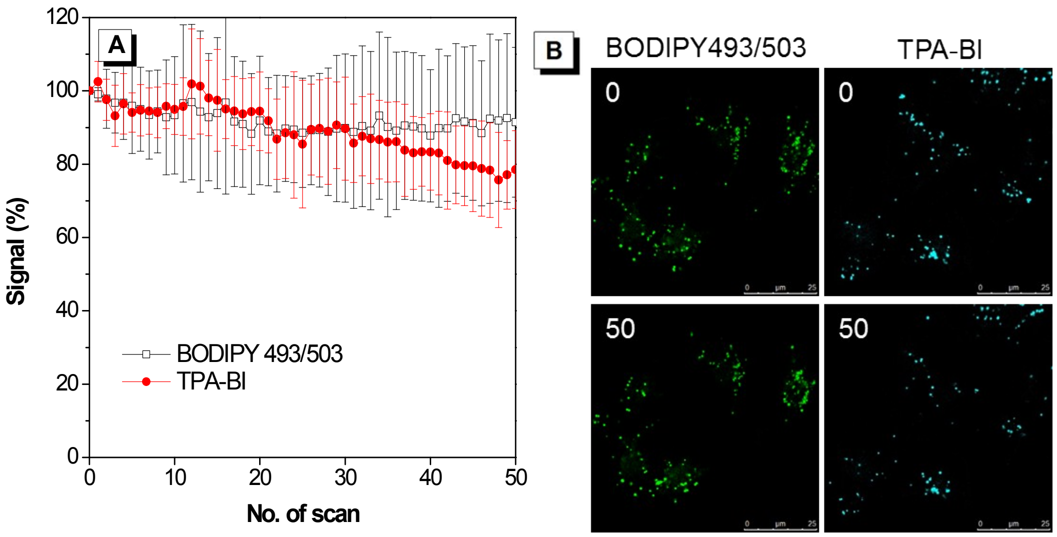

Fig. S11 (A) Change in fluorescence from HeLa cells stained with 5 μM of TPA-BI or 5 μM of BODIPY 493/503 upon continuously scanning by laser light. The time for each scan was 5.24 s. (B) Confocal images of HeLa cells at scan 0 and scan 50.

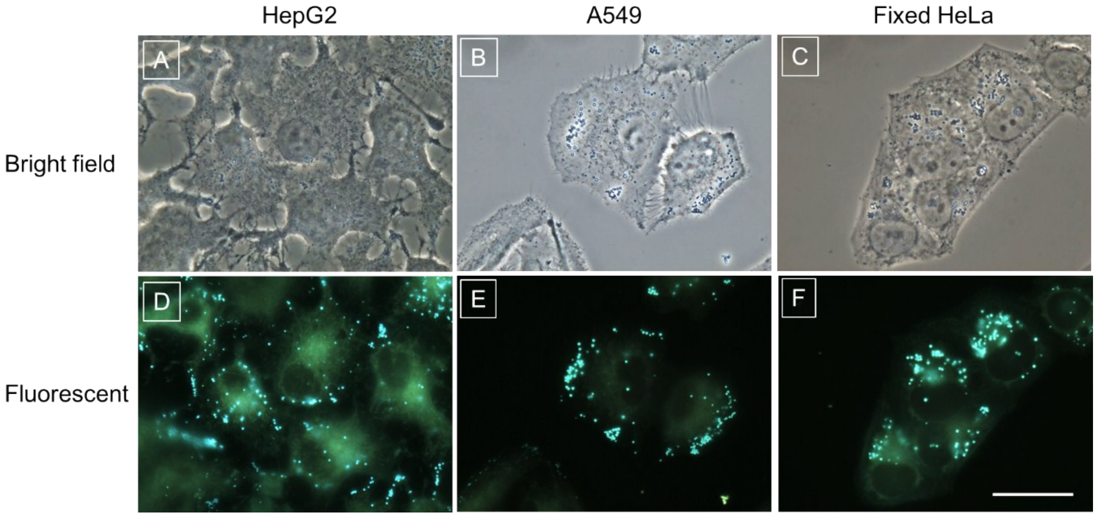

Fig. S12 (A–C) Bright field and (D–F) fluorescent images of (A) HepG2 (B) A549 and fixed HeLa cells stained with TPA-BI for 15 min. Concentration = 1 μM; λ_{ex} = 400–440 nm; scale bar: 20 μm.

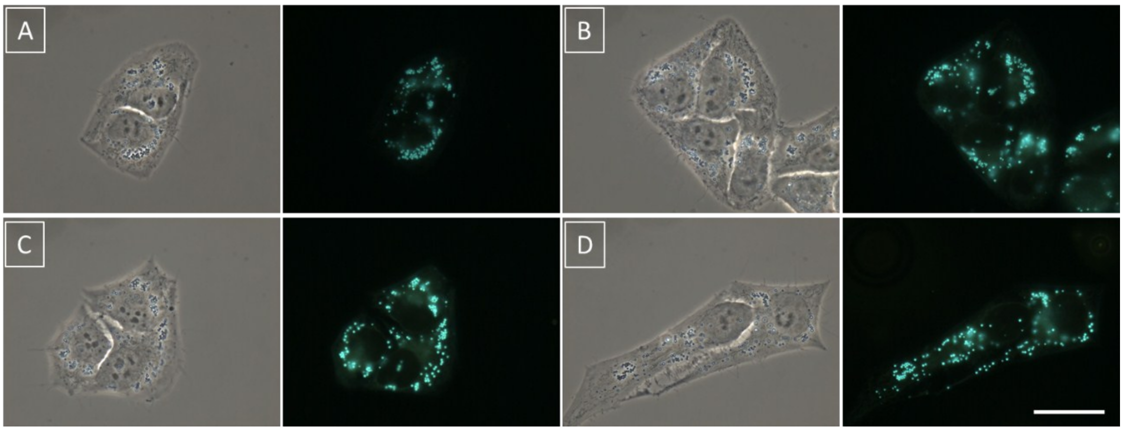

Fig. S13 Fluorescent microscope images of lipid droplets in HeLa cells stained with various concentrations of TPA-BI for 15min. (A) 1 μM, (B) 2 μM, (C) 5 μM and (D) 10 μM. HeLa cells were pretreated with 50 μM oleic acid for 5.5 h. Scale bar: 30 μm.

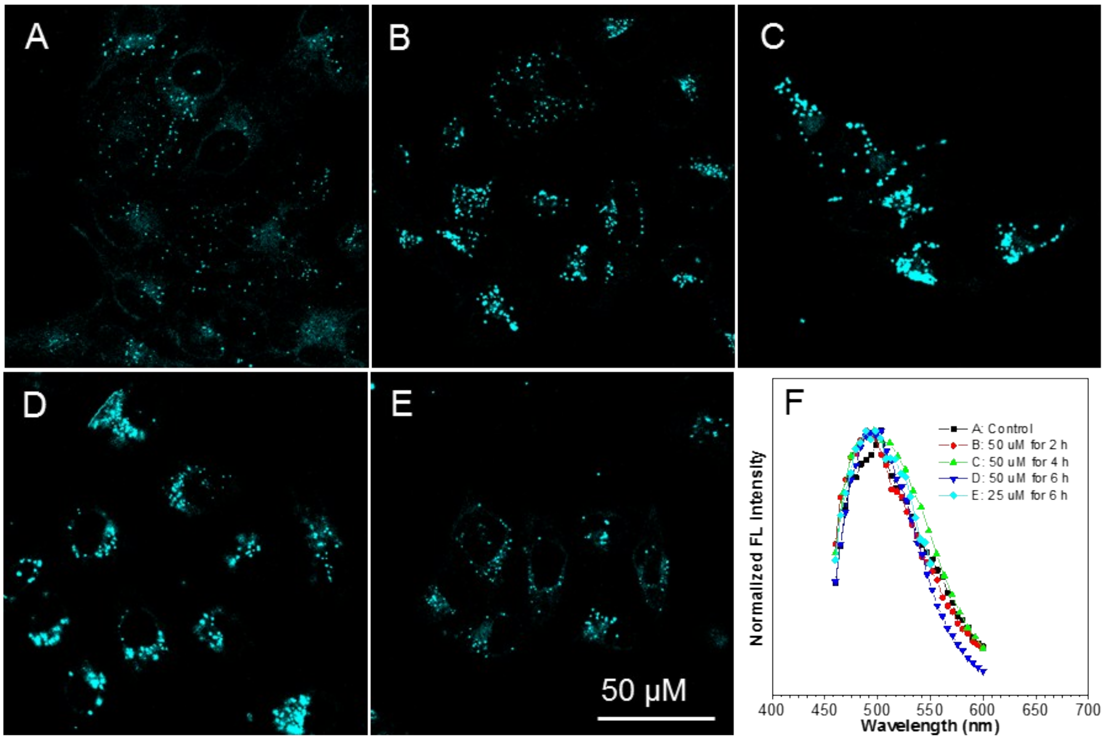

Fig. S14 (A-E) Confocal images of lipid droplets in HeLa cells stained with 5 μM of TPA-BI for 30 min. (A) HeLa cells without oleic acid treatment as the control group. HeLa cells were pretreated with 50 μM oleic acid for (B) 2 h, (C) 4 h and (D) 6 h. (E) HeLa cells were pretreated with 25 μM oleic acid for 6 h. (F) *In situ* fluorescence spectra of lipid droplets in HeLa cells of A–E recorded by confocal microscope. Scale bar: 50 μm.

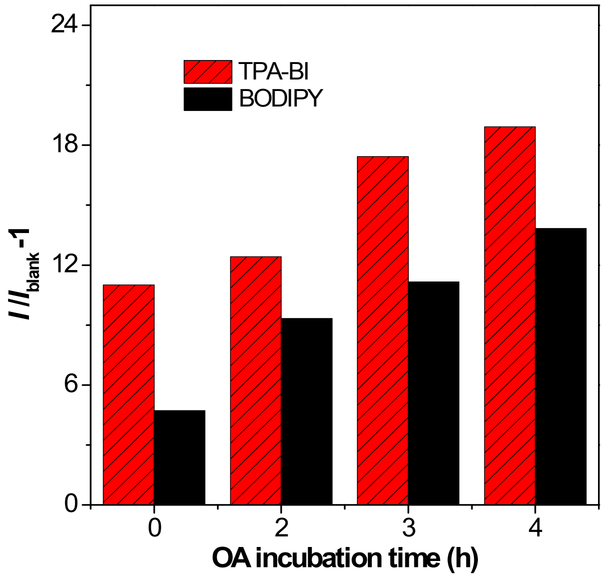

Fig. S15 Lipid droplet qualitative analysis by flow cytometry: the HeLa cells were pretreated with 50 μM of oleic acid for 0, 2, 3 and 4 h and then stained with 1μM of TPA-BI or 1μg/mL of BODIPY 493/503. 10000 events were measured.

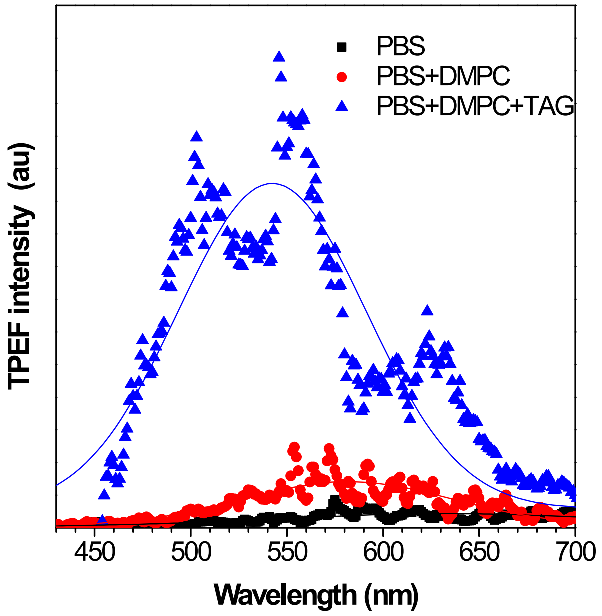

Fig. S16 Fluorescence spectra of 20 μM of TPA-BI in PBS, PBS with DMPC, and PBS with DMPC and TAG excited at 840 nm. The spectra were averaged from three measurements using Gaussian single peak fitting. The peak intensity was 6, 15 and 107 arbitrary unit, respectively.

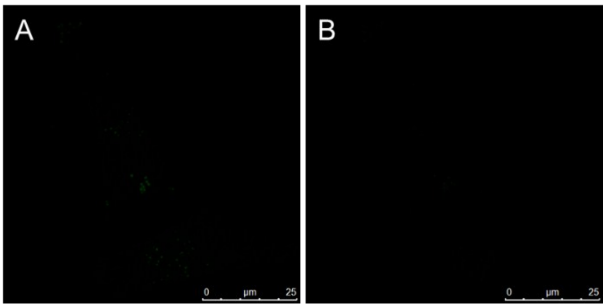

Fig. S17 2PM fluorescent images of HeLa cells stained with 5 μM of BODIPY 493/503 for 20 min and excited at (A) 900 and (B) 980 nm.
